# Dilated Perivascular Space in the Midbrain May Reflect Dopamine Neuronal Degeneration in Parkinson’s Disease

**DOI:** 10.3389/fnagi.2020.00161

**Published:** 2020-06-05

**Authors:** Yanxuan Li, Zili Zhu, Jie Chen, Minming Zhang, Yunjun Yang, Peiyu Huang

**Affiliations:** ^1^Department of Radiology, The First Affiliated Hospital of Wenzhou Medical University, Wenzhou, China; ^2^Department of Radiology, The Second Affiliated Hospital, Zhejiang University School of Medicine, Hangzhou, China

**Keywords:** perivascular space, Parkinson’s disease, dopamine transporter imaging, tau protein, midbrain, basal ganglia

## Abstract

**Background:** The imbalance between the production and clearance of alpha-synuclein and its consequent accumulation plays a pivotal role in the pathogenesis of Parkinson’s disease (PD). The diminished clearance of alpha-synuclein may be partly attributable to impaired interstitial fluid, which can be reflected by the extent of dilated perivascular space (dPVS). We studied the association between dPVS and dopamine neuronal degeneration.

**Method:** We screened 71 healthy controls (HCs) and 88 patients from the Parkinson Progression Markers Initiative (PPMI) database. The dPVS was evaluated in different brain regions on axial T2-weighted images, and dopamine transporter (DAT) imaging data was used to elucidate the extent of dopaminergic neuronal degeneration. Patients with PD were further divided into two groups (SN + PD and SN − PD groups) according to whether dPVS was observed in the substantia nigra (SN). DAT uptake values and clinical scales were compared between the patients with PD and HCs and against dPVS scores. We also investigated the correlation between baseline dPVS scores and longitudinal DAT changes.

**Results:** Relative to the HCs, patients with PD had more dPVS in the SN and basal ganglia (BG). PD patients with dPVS in the SN region exhibited greater expression of tau protein in cerebrospinal fluid (*P* = 0.038) and a trend towards decreased DAT binding (*P* = 0.086) relative to those without SN dPVS. No correlations were found between dPVS scores and DAT uptake values or between dPVS scores and longitudinal DAT changes.

**Conclusion:** The dPVS in the SN of patients with PD may reflect the degeneration of dopaminergic neurons.

## Introduction

The second-most common neurodegenerative disease among older adults, Parkinson’s disease (PD) manifests as various motor and non-motor symptoms primarily attributable to the excessive accumulation of toxic alpha-synuclein and a series of secondary alterations (de Lau and Breteler, 2006; Suchowersky et al., 2006; Narayanan et al., 2013; Chung et al., 2016; Huang et al., 2016; Sundaram et al., 2019; Li et al., 2020). Hence, the imbalance between the production and clearance of alpha-synuclein (Bobela et al., 2015; Abeliovich and Gitler, 2016) plays a pivotal role in PD development (Webb et al., 2003; Ebrahimi-Fakhari et al., 2011; Maiti et al., 2017).

Several studies have shown that diminished drainage mediated by the brain glymphatic system might contribute to the impaired extracellular clearance of soluble alpha-synuclein and aggravate PD pathology. Recently, Zou et al. (2019) ligated deep cervical lymph nodes in A53T mice at 18 weeks of age and thus blocked meningeal lymphatic drainage. Six weeks later, decreases in cerebrospinal fluid flow were observed along with depositions of alpha-synuclein and losses of astroglial aquaporin-4 (AQP-4) in the substantia nigra (SN). These findings strongly suggest that the dysfunction of the glymphatic system aggravates the accumulation of alpha-synuclein and further accelerates the loss of dopaminergic neurons and motor functionality thereby.

Imaging studies of the human brain also support this theory. Detectable with T2-weighted magnetic resonance (MR) images, perivascular spaces—an important component of the brain glymphatic system that functions as a conduit to remove soluble proteins and toxic metabolites from the brain (Aspelund et al., 2015; Mezey and Palkovits, 2015; Ramirez et al., 2015; Louveau et al., 2016)—dilate to restore drainage functionality following the stacking of metabolite wastes and cell debris therein (Mestre et al., 2017).

Prior research has found that dilated perivascular spaces (dPVS) in the basal ganglia (BG) might be related to cognitive decline and tremor in patients with PD (Park et al., 2019; Shibata et al., 2019; Wan et al., 2019). However, other research has failed to identify such associations (Kim et al., 2018), and their veracity thus remains uncertain. Importantly, whether dPVS in the midbrain (Elster and Richardson, 1991; Papayannis et al., 2003; Ferrer et al., 2017) are associated with the loss of dopaminergic neurons in the SN of patients with PD remains unclear.

The present study sought to ascertain whether dPVS is associated with the degeneration of the midbrain dopaminergic systems and the clinical symptoms of patients with PD. By analyzing the correlation between dPVS scores (evaluated on T2-weighted MR images) and dopamine transporter (DAT) uptake values obtained from both cross-sectional and longitudinal data, as well as the correlation between PVS scores and motor/non-motor scales, we explore the hypothesis that dPVS is related to dopamine neuron damage by diminishing DAT uptake and exacerbating clinical symptoms.

## Materials and Methods

### Participants

**Figure 1:**
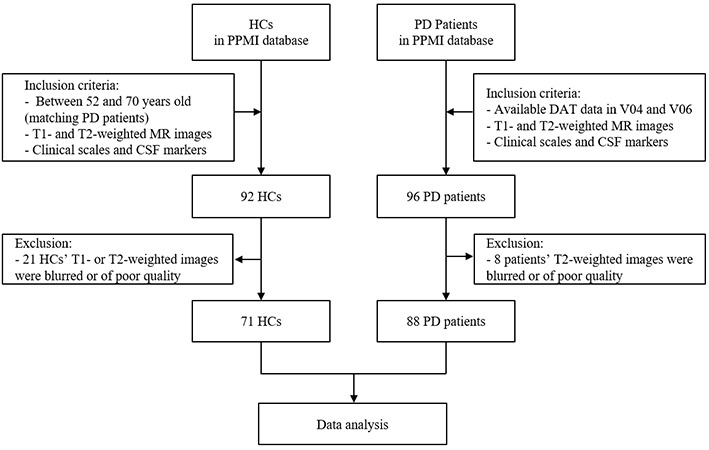
Subject screening workflow.

Participant data were obtained from the Parkinson Progression Markers Initiative (PPMI) database^1^. The PPMI study was approved by the institutional review board of all participating sites and written informed consent was obtained from all participants before their registration in the study. Since data obtained from the DAT scans were most complete at the V04 and V06 time points, we used V04 as the baseline and V06 as the 1-year follow-up. A total of 96 patients with PD were included; both baseline and follow-up T1- and T2-weighted MR images, as well as DAT scan data, were available for all of them. Due to the T2 images of eight patients have been blurry, they were excluded from the study. Nighty-two healthy controls (HCs; age range, 52–70 years) with T1- and T2-weighted MR images and clinical scale records were collected from the PPMI database. Because the T1- or T2-weighted MR images of 21 HCs were blurred or of poor quality, they were excluded from the study (Figure 1). The MR images of all participants were obtained from multiple research centers using the ADNI MRI protocol, a detailed report of which is available from the ADNI website^2^.

### Assessment

Clinical evaluations and DAT results at baseline and 1-year follow-up were available for all 88 patients with PD; data concerning cerebral spinal fluid (CSF) markers [Aβ 42, total tau, phosphorylated tau 181P (p-Tau181P) and Alpha-synuclein] and systolic and diastolic blood pressure in the supine position were only collected in this population at baseline. Clinical scale scores and the expression of CSF markers, but not DAT results, were available for the 71 HCs. All examinations and assessments are reported in detail at http://www.ppmi-info.org/study-design. The motor symptoms of patients were assessed with Unified Parkinson’s Disease Rating Scale Part III (UPDRS-III). The following clinical scales were further used in this study: Geriatric Depression Scale (GDS), Epworth Sleepiness Scale (ESS), and Montreal Cognitive Assessment (MoCA).

**Figure 2:**
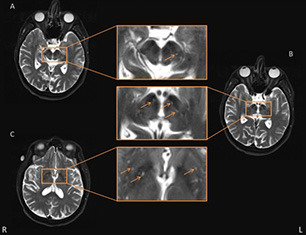
Representative axial T2-weighted images showing basal ganglia (BG) and midbrain perivascular space (PVS). Some typical PVS are indicated by the orange arrow. Panels **(A,B)** are two consecutive layers showing PVS in the midbrain, in which the PVS in panel **(A)** is continuous to the rightmost PVS in panel **(B)**, and the PVS is shown passing through the SN. The other PVS in panel **(B)** were also deemed adjacent to SN. Panel **(C)** shows the axial slice which had the most PVS in the BG area.

The mean of the bilateral DAT uptake values (Ave-DAT) was calculated for the caudate and putamen nuclei for further analysis. The dPVS were evaluated on axial T2-weighted images according to the Standards for Reporting Vascular Changes on Neuroimaging criteria (Wardlaw et al., 2013) by two trained neuroradiologists (YL and ZZ, with 4 and 2 years of experience, respectively) blinded to the patients’ clinical information and the other evaluator’s scoring. Briefly, the dPVS were defined as structures situated perpendicular to the surface of the brain in the direction of perforating vessels that evinced cerebrospinal fluid signals and presented sharp edges of less than 3 mm in diameter. Hence, they were round or ovoid on axial slices (in the BG) and linear on longitudinal slices [in the centrum semiovale (CSO)]. The dPVS corresponded to high signals on T2-weighted images and low signals on T1-weighted and FLAIR images. This method requires the differentiation of dPVS from lacunes, which are generally oval-shaped lesions of a diameter larger than 3 mm. The dPVS in the BG and CSO were assessed with a 4-point visual rating scale (0 = absent dPVS, 1 = less than 10 dPVS, 2 = 11–20 dPVS, 3 = 21–40 dPVS, 4 = more than 40 dPVS) according to the method described by Doubal et al. (2010). After reviewing all relevant slices, the dPVS were counted in the slice that featured the highest number of dPVS. Each hemisphere was assessed separately, and the hemisphere with a higher score was used. The evaluators also counted the number of dPVS in the midbrain and thalamus to ascertain the presence of dPVS in the SN. A dPVS that continued across multiple slices was counted only once. The presence of dPVS in the SN was confirmed if the dPVS was located in or connected to the SN. The boundaries of the regions of interest were defined by comparison to standard brain atlases. Figure 2 illustrates a representative case of a patient with PD. The inter-rater reliability was good for the BG-PVS score (intraclass correlation coefficient = 0.75) and the number of midbrain PVS (intraclass correlation coefficient = 0.80). The PVS rating scale of the senior radiologist was used for the analysis.

### Statistical Analysis

Demographic data, expression of CSF markers, imaging characteristics, and clinical scales were compared between the HCs and patients with PD. Two-sample *t*-tests were used for continuous data, while Wilcoxon Rank-Sum tests were used for categorical data. Because the loss of dopaminergic neurons in the SN of the midbrain is particularly important to the development of PD, we divided the 88 patients with PD into two groups according to the presence of dPVS in the SN region: 40 PD patients had dPVS in the SN region (SN + PD), and 48 PD patients did not (SN − PD). Baseline clinical scales, CSF markers, Ave-DAT values, and imaging characteristics were compared between the two groups. Subsequently, after controlling for age, sex, and level of education, we calculated the partial Pearson’s correlation coefficients between baseline dPVS scores/numbers and Ave-DAT, clinical scales, and CSF markers. Finally, we examined the correlation between baseline dPVS scores and the rate of longitudinal changes of Ave-DAT and other clinical scales. To calculate the rate of change, we took the difference between the baseline and follow-up values divided by the absolute baseline value.

## Results

The demographic characteristics of the patients with PD are presented in Table 1. Age (*p* = 0.298), sex (*p* = 0.830), and years of education (*p* = 0.064) did not differ significantly between the HCs and patients with PD. There were significant differences in the dPVS of the SN (*p* = 0.022) and BG (*p* < 0.001) between patients with PD and HCs. Differences between the two populations were also found in p-Tau181P expression (*p* = 0.002), MoCA score (*p* < 0.001), and GDS score (*p* = 0.002).

**Table 1:** Clinical and demographic data of participants and comparisons.

Parameters	SN + PD		SN − PD		HC		*p*-value	
	M	SEM	M	SEM	M	SEM	SN + PD vs. SN − PD	PD vs. HC
Sex(M/F)	32/15		24/17		44/27		0.356^#^	0.830^#^
Age	62.06	1.135	60.9	1.614	60.31	0.635	0.558	0.294
Years of education	15.6	0.39	14.9	0.487	16.1	0.312	0.265	0.064
Duration of PD	1.72	0.103	1.64	0.114	−	−	0.6	−
Ave-DAT (caudate)	1.694	0.684	1.821	0.059	−	−	0.173	−
Ave-DAT (putamen)	0.657	0.029	0.737	0.036	−	−	0.086	−
SN dPVS (+/−)	47		41		25/46		−	0.022^#^
Midbrain-dPVS number	3.6	0.275	0.88	0.189	1.86	0.291	<0.001	0.194
BG-dPVS score	1.87	0.078	1.76	0.076	1.35	0.07	0.294	<0.001
Thalamus-dPVS number	1.81	0.254	1.66	0.246	2.35	0.294	0.674	0.064
CSO-dPVS score	3.45	0.135	3.37	0.12	3.01	0.118	0.659	0.008
Systolic blood pressure (mmHg)	127.83	2.267	130.39	3.069	−	−	0.497	−
Diastolic blood pressure (mmHg)	75.15	1.281	76.83	1.853	−	−	0.449	−
Aβ 42 (pg/ml)	380.682	13.798	379.146	14.808	400.528	13.69	0.94	0.218
p-Tau181P (pg/ml)	15.16	1.298	14.341	1.315	19.989	1.39	0.66	0.002
Total tau (pg/ml)	47.791	2.924	40.098	2.191	48.524	2.342	0.038	0.15
Alpha-synuclein (pg/ml)	1,895.191	103.57	1,783.61	110.123	2,021.102	95.068	0.462	0.139
MoCA	26.68	0.45	26.61	0.392	28.14	0.138	0.907	<0.001
UPDRS-III	23.53	1.525	22.15	0.869	−	−	0.564	−
ESS	6.17	0.604	6.39	0.62	5.59	0.402	0.8	0.258
GDS	2.23	0.363	2.59	0.463	1.25	0.221	0.547	0.002

*^#^Is for the Wilcoxon Rank-Sum test, the others were t-tests. Abbreviation: SN, substantia nigra; PD, Parkinson’s disease; M/F, male/female; DAT, dopamine transporter; dPVS, dilated perivascular space; BG, basal ganglia; CSO, centrum semiovale; UPDRS-III, Unified Parkinson’s Disease Rating Scale Part III; GDS, Geriatric Depression Scale; ESS, Epworth Sleepiness Scale; MoCA, Montreal Cognitive Assessment; p-Tau181P, phosphorylated tau 181P*.

There were no significant differences in age (*p* = 0.558), sex (*p* = 0.356), and years of education (*p* = 0.265) between the SN + PD and SN − PD groups; however, they differed significantly in their total expression of tau-protein (*p* = 0.038) and many midbrain dPVS (*p* < 0.001). While we found that the Ave-DAT (putamen) of the SN + PD group was lower than that of the SN − PD group, the corresponding *p*-value was only marginally significant (*p* = 0.086). Other statistical results were nonsignificant (Table 1).

No correlations were found between the dPVS scores and baseline CSF markers or between the dPVS scores and cognitive scales (Tables s 2, 3). Correlations between dPVS scores and longitudinal alterations were nonsignificant. The results remained nonsignificant after Bonferroni correction.

**Table 2:** Correlations between midbrain or BG-d PVS number and indicators (baseline).

Parameters	Midbrain-dPVS number			BG-dPVS score		
	*R*	*P*-value^a^	*P*-value^b^	*R*	*P*-value^a^	*P*-value^b^
Ave-DAT (caudate)	0.09	0.41	1	−0.13	0.25	1
Ave-DAT (putamen)	0.1	0.36	1	−0.02	0.87	1
Systolic blood pressure (mmHg)	−0.148	0.177	1	0.145	0.159	1
Diastolic blood pressure (mmHg)	−0.154	0.186	1	0.016	0.882	1
Aβ 42 (pg/ml)	0.02	0.89	1	0.05	0.64	1
p-Tau181P (pg/ml)	0.05	0.67	1	0.03	0.78	1
Total tau (pg/ml)	0.09	0.43	1	−0.07	0.5	1
Alpha-synuclein (pg/ml)	−0.13	0.23	1	0.15	0.18	1
UPDRS-III	0.03	0.78	1	−0.03	0.76	1
MoCA	0.14	0.21	1	−0.05	0.65	1
ESS	−0.1	0.36	1	−0.03	0.8	1
GDS	−0.14	0.2	1	0.11	0.31	1

*^a^Unadjusted; ^b^after Bonferroni correction. Abbreviation: dPVS, dilated perivascular space; BG, basal ganglia; DAT, dopamine transporter; UPDRS-III, Unified Parkinson’s Disease Rating Scale Part III; GDS, Geriatric Depression Scale; ESS, Epworth Sleepiness Scale; MoCA, Montreal Cognitive Assessment; p-Tau181P, phosphorylated tau 181P*.

**Table 3:** Correlations between altered midbrain or BG-d PVS number and indicators (longitudinal).

Parameters	Midbrain-dPVS number			BG-dPVS score		
	*R*	*P*-value^a^	*P*-value^b^	*R*	*P*-value^a^	*P*-value^b^
DAT ratio (caudate)	0.03	0.81	1	−0.03	0.82	1
DAT ratio (putamen)	−0.03	0.81	1	−0.16	0.15	1
Aβ 42 (pg/ml)	0.02	0.87	1	0.05	0.64	1
p-Tau181P (pg/ml)	0.05	0.67	1	0.03	0.78	1
Total tau (pg/ml)	0.09	0.43	1	−0.07	0.5	1
Alpha-synuclein (pg/ml)	−0.13	0.23	1	0.15	0.18	1
MoCA ratio	−0.11	0.32	1	0.09	0.41	1
UPDRS-III ratio	−0.06	0.59	1	−0.07	0.53	1
ESS ratio	−0.03	0.8	1	−0.01	0.9	1
GDS ratio	0.14	0.22	1	−0.08	0.08	1

*^a^Unadjusted; ^b^after Bonferroni correction. Abbreviation: dPVS, dilated perivascular space; BG, basal ganglia; DAT, dopamine transporter; UPDRS-III, Unified Parkinson’s Disease Rating Scale Part III; GDS, Geriatric Depression Scale; ESS, Epworth Sleepiness Scale; MoCA, Montreal Cognitive Assessment; p-Tau181P, phosphorylated tau 181P*.

## Discussion

The present study found that PD patients had more dPVS in the BG and SN and lower expression of p-Tau181P relative to HCs. Further, although PD patients with SN dPVS were found to exhibit lower DAT uptake and higher levels of CSF tau, the difference in DAT uptake was only marginally significant. Correlation analyses and longitudinal observations showed no significant associations between dPVS, DAT, and clinical symptoms. These results suggest a mechanistic link between dPVS and SN degeneration.

We found that dPVS in the BG differed significantly between HCs and PD patients. Our findings are consistent with those of previous studies, suggesting that the appearance of these signs may reflect certain features of PD pathology rather than normal aging. Some studies have found that the appearance of dPVS in patients with PD is related to their clinical symptoms: Wan et al. (2019) found that dPVS in the BG correlated with tremor, and Schwartz et al. (2012) observed that characteristics of the small-vessel disease, including dPVS, might be associated with motor impairments in PD. However, such correlations were not stable. It is possible that brain alterations outside the BG area have imposed stronger influence over clinical symptoms, therefore, we found no significant correlation between BG dPVS and clinical symptoms.

**Figure 3:**
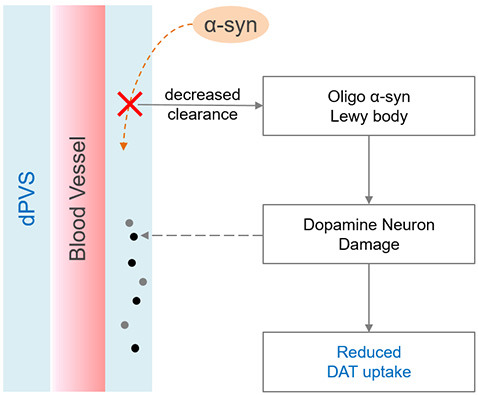
Possible mechanisms linking dilated PVS (dPVS) and reduced dopamine transporter (DAT) uptake. On one hand, decreased alpha-synuclein clearance may accelerate the formation of Lewy body, and lead to dopamine neuron damage. On the other hand, dopamine neuron degeneration can produce excessive metabolic wastes, which may stack in the perivascular space and cause compensatory dilation.

We further observed PD patients have more dPVS near the SN relative to HCs, a result not previously reported in the literature. Additionally, PD patients with dPVS in the SN showed a trend towards decreased DAT uptake (*p* = 0.086) when compared to their counterparts without SN dPVS. This observation suggests a link between SN dPVS and dopaminergic neuronal degeneration. The mechanisms underlying such association might be complex (Figure 3): during brain aging, hypertension and the loss of AQP-4 water channels could impair the drainage of local interstitial fluid, and disrupt alpha-synuclein clearance (Peng et al., 2016; Lenck et al., 2018), for which the perivascular spaces function as an important pathway (Zou et al., 2019). Accumulated alpha-synuclein may transform into oligo alpha-synuclein and Lewy bodies (Lashuel et al., 2013) that could damage dopaminergic neurons and reduce DAT uptake. Furthermore, multiple genetic and environmental factors might cause the degeneration of SN dopaminergic neurons, yielding excessive neuronal debris as well as other metabolic waste and cell fragments (Zou et al., 2019). Large molecules might consequently stack in the perivascular spaces, inducing compensatory dilation. Additionally, the loss of neurons and glial cells could also shrink local brain tissues, creating larger spaces between blood vessels and brain parenchyma—i.e., dPVS. Importantly, the aforementioned processes might interact with one another, creating a cycle that causes escalating damage to the SN.

We found that patients with SN dPVS exhibited more total tau expression than did those without SN dPVS, suggesting that PD patients with SN dPVS suffer from a higher degree of neuron degeneration. Tau is a microtubule-associated protein, which may be released into extracellular spaces during the course of several neurodegenerative diseases. Interestingly, Alpha-synuclein could accelerate tau phosphorylation, and their interaction may induce the fibrilization of both proteins. Tau pathologies may compromise axonal transport and cause the further aggregation of alpha-synuclein within the cell body (Giasson et al., 2003; Lei et al., 2010). Although the p-Tau181P is considered more harmful than total tau, elevated concentrations of the latter have also been found in Alzheimer’s disease patients (Hampel et al., 2008). Despite these mechanistic links, several studies investigating the role of CSF tau in PD pathology yielded conflicting results (Loeffler et al., 2019). However, in agreement with previous results (Kang et al., 2013; Parnetti et al., 2014), the present study observed the CSF total tau concentration in HCs to exceed that of patients with PD. This phenomenon may be due to the interaction between tau proteins and alpha-synuclein, which may limit tau-protein release into the CSF. The role of CSF tau in PD pathogenesis thus requires further investigation.

To elucidate the causal relationship between dPVS and dopamine neuron degeneration, we performed a correlation analysis between baseline dPVS scores and follow-up Ave-DAT but found no significant correlation. However, this nonsignificant finding does not imply the lack of a causal relationship between dPVS scores and Ave-DAT. The degeneration may have been insufficient across the relatively short duration of follow-up to reflect the contribution of dPVS. The limited imaging resolution was also a major constraint in conducting analyses of dPVS. The state-of-art sub-millimeter isotropic T2 imaging method (Gao et al., 2014) may be used in the future to better facilitate dPVS quantification.

## Conclusions

Our findings indicate that PD patients had more dPVS in the SN compared to HCs, a discrepancy may relate to the degeneration of dopaminergic neurons in the SN. However, because the decrease of DAT uptake was only marginally significant (*p* = 0.086), further study is warranted to confirm our findings and validate the potential value of dPVS as a marker for PD pathology.

## Data Availability Statement

The datasets analyzed for this study can be found in the PPMI data repository (http://www.ppmi-info.org/access-data-specimens/download-data/).

## Ethics Statement

The studies involving human participants were reviewed and approved by the PPMI study, which was approved by the institutional review board of all participating sites. The patients/participants provided their written informed consent to participate in this study.

## Author Contributions

PH, MZ and YY direct the experiment’s overall design and revised the manuscript. YL and ZZ evaluated the image data. YL and JC conceived the design details and analytic plan, then performed statistical analyses and drafted the manuscript.

## Conflict of Interest

The authors declare that the research was conducted in the absence of any commercial or financial relationships that could be construed as a potential conflict of interest.

## Footnotes

^1^www.ppmi-info.org/data

^2^http://adni.loni.usc.edu/methods/documents/mri-protocols/
